# Morpho-phonemic analysis boosts word reading for adult struggling readers

**DOI:** 10.1007/s11145-017-9774-9

**Published:** 2017-09-23

**Authors:** Susan H. Gray, Linnea C. Ehri, John L. Locke

**Affiliations:** 1 Communication Sciences and Disorders, Bridgewater State University, Bridgewater, MA 02325 USA; 2 Educational Psychology, CUNY Graduate Center, 365 Fifth Avenue, New York, NY 10016 USA; 3 Department of Speech-Language-Hearing Sciences, Lehman College, CUNY, 250 Bedford Park Boulevard West, Bronx, NY 10468 USA

**Keywords:** Reading disabilities, Morphological awareness, Reading intervention, Adult literacy, Vocabulary instruction

## Abstract

A randomized control trial compared the effects of two kinds of vocabulary instruction on component reading skills of adult struggling readers. Participants seeking alternative high school diplomas received 8 h of scripted tutoring to learn forty academic vocabulary words embedded within a civics curriculum. They were matched for language background and reading levels, then randomly assigned to either morpho-phonemic analysis teaching word origins, morpheme and syllable structures, or traditional whole word study teaching multiple sentence contexts, meaningful connections, and spellings. Both groups made comparable gains in learning the target words, but the morpho-phonemic group showed greater gains in reading unfamiliar words on standardized tests of word reading, including word attack and word recognition. Findings support theories of word learning and literacy that promote explicit instruction in word analysis to increase poor readers’ linguistic awareness by revealing connections between morphological, phonological, and orthographic structures within words.

## Introduction

Adult struggling readers are a heterogeneous population with vastly different reading skill levels and language learning profiles. They may have difficulties in any component of literacy, including decoding (word attack), word reading (word recognition), spelling, vocabulary and reading comprehension (Alamprese, MacArthur, Price, & Knight, 2011; Ehri, 1997; Sabatini, 2002). This preliminary intervention study addresses the diverse needs of adult struggling readers by teaching analysis of complex vocabulary words to adults seeking alternative high school diplomas or General Education Diplomas (GED) with sixth grade reading skills on average. Vocabulary instruction involving analysis of words’ morphemes (smallest units of meaning) and phonemes (smallest units of sound) is compared to a more traditional kind of vocabulary instruction involving the study of whole words.

Twenty percent of American public high school students drop out (National Center for Education Statistics, 2015) often with low literacy skills and few opportunities for meaningful employment (Wayman, 2001) and civic engagement (Levinson, 2012). In one study of GED students, up to half of them read below the fifth grade level, and about forty percent of those between 16 and 20 years old had learning disabilities or attention deficit disorders (Perin, Flugman, & Spiegel, 2006).

Many students in adult education programs have reading difficulties consistent with dyslexia (Greenberg, Wise, Morris, Fredrick, Rodrigo, Nanda & Pae, 2011), a severe reading disability characterized by reduced decoding and spelling skills with poor phonological awareness, awareness of words’ sound structures (Stanovitch & Siegel, 1994) that persists through the lifespan (Shaywitz & Fletcher, 1999). Adults with reading disabilities tend to have uneven component reading skills, performing better on tests of word reading than on tests of phonological and decoding skills (Greenberg, Ehri, & Perin, 1997; Sabatini, Sawaki, Shore, & Scarborough, 2010). For example, when adult struggling readers were matched with typical readers in third to fifth grade, they outperformed the children on sight word reading tasks, but performed more poorly on tasks of decoding, sound deletion and phoneme segmentation, indicating severe phonological deficits in reading disabled adults (Greenberg et al., 1997).

### Theories of word learning that connect morphemes, phonemes and spellings

Several theories of word learning assert that teaching connections between words’ morphemes, phonemes and spellings will increase poor readers’ component literacy skills. Chomsky (1970) promoted the teaching of “lexical spellings” to draw readers’ attention to the ways in which English orthography reveals deep semantic relationships between morphological relatives, such as words that share the same root or base morphemes, despite superficial alternations in pronunciations (e.g., *precise* and *precision* have related meanings preserved in their shared base word spellings, despite the vowel and consonant pronunciation shifts). Similarly, Ehri (e.g., 1978, 1999, 2005) theorized that readers must analyze words’ multi-linguistic identities (e.g., semantic, phonological and orthographic structures) in order to amalgamate those identities into a single orthographic image stored in the lexicon, an image whose linguistic identities have been fully analyzed, thereby enabling the reader to recognize the word instantly, by sight. In her theory of sight word learning, the mapping of words’ meaning, sound and spelling structures results in accurate, automatic reading of individual words (sight words), the hallmark of skilled reading. Sight word reading develops through phases of increasing awareness of linguistic connections within words, in which “the consolidated alphabetic phase replaces the full alphabetic phase when the predominant types of connections for retaining sight words are morphographic” (Ehri, 2005, p. 150).

In the Lexical Quality Hypothesis, Perfetti and Hart (2001, 2002) also view the role of precise word knowledge and fluent word reading as central to literacy learning. Poor readers lack high quality internal representations of words called lexical representations. Readers have high quality representations when they know precisely words’ spellings, meanings, and pronunciations, and are able to read them fluently. Readers have low quality representations when they have not precisely specified words’ orthographic, semantic, and phonological structures. Poor ability to analyze connections between words’ spellings, meanings, and pronunciations obstructs fluent word level reading. Low quality lexical representations subsequently impede reading comprehension, which depends upon accurate, fluent word recognition (Perfetti, 2007). Thus, the Lexical Quality Hypothesis views proficient reading as a nested process, in which higher level reading skills, like vocabulary and comprehension, depend upon lower level reading skills, like word analysis and word recognition. In support of this hypothesis, there is evidence that complex word identification serves as a moderator between morphological awareness and reading comprehension for fifth graders with poor multi-syllabic word reading skills (Gilbert, Goodwin, Compton, & Kearns, 2014).

### The importance of vocabulary for older struggling readers

Vocabulary difficulties are often seen in minority language learners, who comprise a large proportion of students in adult learning programs (e.g., Perin et al., 2006). English Language Learners tend to perform substantially below average on vocabulary tests (Proctor, Carlo, August, & Snow, 2005; Swanson, Saez, & Gerber, 2006), and so do students from minority language backgrounds who may speak a dialect of English, such as African American English or Chicano English. In fact, native English speakers from minority language backgrounds perform only slightly better than English Language Learners on tests of vocabulary (Beech & Keys, 1997; Droop & Verhoeven, 2003; Hutchinson, Whitely, Smith, & Connors, 2003; Leseman & de Jong, 1998; Verhoeven, 2000). Thus, vocabulary instruction is a crucial element in the literacy needs of minority language learners, including both English Language Learners and native English speakers of minority dialects.

Literacy achievement depends on accurate comprehension of complex vocabulary in academic language, which is “the specialized language, both oral and written, of academic settings that facilitates communication and thinking about disciplinary content” (Nagy & Townsend, 2012, p. 92). Academic vocabulary words are typically morphologically complex, with base words and multiple affixes, such as derivational suffixes (e.g., *environmental*, whose base word *environ* has two derivational suffixes, *ment* and *al*). They include both general terms used within academic settings (e.g., *hypothetical*) and specific terms residing within certain disciplines (e.g., *Reconstruction* in Amercian History) (Nagy & Townsend, 2012). In one instructional program called Word Generation, 697 middle school students were taught 5 general academic vocabulary words each week, within highly motivating topics and activities in math, social studies, and English, resulting in medium treatment effect sizes on measures of word knowledge (Snow, Lawrence & White, 2009). Although there is a paucity of research on vocabulary interventions for minority language learners, one such study provided 18 weeks of a program called Academic Language Instruction for All Students (ALIAS) to 476 sixth graders in English Language Arts classrooms. Eight or nine general academic vocabulary words per unit were taught over 18 weeks, using engaging texts with activities in reading, writing and morphology. Results showed significant gains in vocabulary knowledge and morphological awareness (awareness of morphemes) for both language minority learners and native English speaking peers (Lesaux, Kieffer, Faller, & Kelley, 2010).

Although the importance of teaching academic vocabulary is well established, most teachers require guidance on how to teach them (Nagy & Townsend, 2012) and students rarely receive high quality vocabulary instruction leading to independent use of new words (Beck, McKeown, & Kucan, 2002; Gersten, Dimino, Jayanthi, Kim, & Santoro, 2010). High quality vocabulary instruction is particularly important for students in low income urban schools, where high proportions of minority language learners face significant obstacles to academic achievement, including teacher shortages, large class sizes, and low student expectations (Anyon, 1980, 1997; Lankford, Loeb, & Wyckoff, 2002). Students who learned English as their second language start school with lower vocabulary skills than their middle income, native English speaking peers (e.g., Cobo-Lewis, Pearson, Eilers, & Umbel, 2002) and the disparity in vocabulary skills widens as they advance in age (Kieffer, 2008; Nakamoto, Lindsey, & Manis, 2007).

### Morphology’s role in literacy

English spelling reflects words’ phonemes, as in letter-sound correspondence (e.g., *link* in which each sound is represented by each letter), and words’ morphemes, as in affixes whose spellings remain stable despite pronunciation changes (e.g., *linked* in which the past tense morpheme is spelled as “ed” despite the */t/* pronunciation). Teaching literacy in ways that increase readers’ awareness of both morphemes and phonemes makes sense given the morpho-phonemic structure of English orthography (Chomsky & Halle, 1968; Venezky, 1999), yet many literacy programs do not include direct teaching of morphemes (Henry, 2003; Nunes & Bryant, 2006).

While it is well established that increasing phonological awareness leads to improved reading skills (Ehri et al., 2001; National Reading Panel, 2000), it is less common knowledge that increasing morphological awareness also improves literacy at all levels of K-12 schooling, especially for reading disabled students (Bowers, Kirby & Deacon, 2010; Goodwin & Ahn, 2010). A meta-analysis of 17 studies of morphological intervention for K-12th graders found moderate treatment effect sizes for overall literacy, phonological awareness, morphological awareness and vocabulary with smaller, still significant, effect sizes for reading comprehension and spelling (Goodwin & Ahn, 2010). One review (Bowers et al., 2010) analyzed the effects of morphological teaching on component literacy skills by considering several linguistic layers of instruction. Drawing from peer-reviewed intervention studies for K-8 students, researchers coded literacy outcomes according to three linguistic layers on which the treatments focused. Morphological teaching at the sub-lexical (within word) layer, such as morphemic analysis of roots and affixes, produced medium to large treatment effect sizes. Morphological instruction at the lexical (word) layer, such as word identification, resulted in medium effect sizes. Morphological training at the supra-lexical (phrases, sentences and discourse) layer, such as reading comprehension, produced only small effect sizes. Thus, focusing on the sub-lexical layer with direct parsing of morphemes within words appeared to produce greater, perhaps more immediate, literacy gains than indirect teaching of morphemes within sentences and passages.

### The need for morpho-phonemic instruction with older readers

Correlational studies reveal close relationships between component literacy skills and readers’ awareness of words’ phonemes and morphemes. In a study investigating the literacy skills of adults in GED, pre-GED and basic education programs, phonological decoding predicted spelling, listening comprehension and reading comprehension. Morphological awareness predicted spelling, listening comprehension and vocabulary, which indirectly predicted reading comprehension. Phonological decoding correlated highly with morphological awareness. Those findings led authors to recommend morphological instruction in adult literacy (Fracasso, Bangs & Binder, 2014). Similarly, in assessments of fifth grade readers who had difficulty reading complex words, the interaction between morphological awareness and complex word reading accounted for significant variance in reading comprehension. Researchers concluded that complex word reading may serve as a mediator between morphological awareness and reading comprehension (Gilbert et al., 2014). Thus, morphological training may promote increased accuracy for complex word reading, and ultimately, better reading comprehension.

Some intervention studies of adolescent and adult struggling readers have reported limited treatment effects (e.g., Greenberg et al., 2011; Sabatini, Shore, Holtzman, & Scarborough, 2011), though novel approaches teaching complex word analysis have shown promise. For example, adults with third to sixth grade reading levels received treatment focused on decoding, fluency, comprehension, extensive reading and combined approaches, yet made relatively limited literacy gains (Greenberg et al., 2011). However, adult poor readers who were taught the morpho-phonemic structure of English orthography (Venezky, 1999), through analysis of words’ meaning, sound, and spelling connections, and use of a meta-cognitive strategy to decode complex words, made greater gains in decoding than those taught using a more traditional approach with a children’s curriculum adapted for adult use (Alamprese et al., 2011). Similarly, adolescent struggling readers who learned to parse complex words into syllables transferred their learning to recognize untaught words, whereas those who learned to read whole words did not demonstrate learning transfer (Bhattacharya & Ehri, 2004).

### Morphological training as compensatory strategy for older struggling readers

Morphological instruction may provide older poor readers who lack phonological and orthographic awareness with a compensatory strategy to increase word recognition, fluent word recognition, thereby enabling access to reading comprehension (Elbro & Arnbak, 1996; Gilbert et al., 2014; Law, Wouters, & Ghesquiere, 2015). Elbro and Arnbak (1996) found that morphological processing may offer a compensatory strategy for Danish adolescents with poor phonological skills. In their first experiment, 15 year olds with dyslexia read compound words faster than non-compound words, and faster reading rates correlated highly with better reading comprehension skills. In their second experiment, adolescents with dyslexia read text faster when it was parsed into morphemes than when it was parsed into syllables, outperforming younger typical normal readers on that task of morphological awareness.

Law et al. (2015) investigated the word reading skills of college adults with dyslexia and results suggested use of morphological compensation in decoding tasks. They divided students with dyslexia into one group who had compensated and another group who had not compensated for their disability. Those who had compensated for dyslexia had histories of poor word reading skills but had advanced their skills to the normal range by the time they were in college, whereas those who had not compensated had poor word reading skills that persisted in college. The compensated group had higher vocabulary skills, so an adjustment was made for vocabulary, before comparing the two groups’ morphological awareness skills. Even after adjusting for the vocabulary difference, morphological awareness skills accounted for 17% of the variance in word reading. Moreover, when morphological awareness was compared with all other literacy measures, its relationship to word reading was the strongest. Authors concluded that adults who had compensated for dyslexia may have forged a new morphological pathway to successful decoding, and noted that “explicit teaching of morphological rules and methods for the morphological decomposition of words could potentially improve adult dyslexics’ morphological awareness, subsequently improving their word reading skills” (Law et al., 2015, p. 269).

While some studies of older struggling readers have provided evidence of morphological compensation to offset poor phonological awareness (e.g., Elbro & Arnbak, 1996; Law et al., 2015), others have shown weaknesses in morphological knowledge. Leong (1999) examined phonological and morphological knowledge in a group of adults who were enrolled at a technical institute and pursuing post-secondary certificate programs in science, technology and health. Students with severe reading disabilities, marked primarily by poor word reading skills, were matched on age and reading level with typical students. Participants completed measures of phonological knowledge, such as oral reading of pseudowords and rhyme matching, and measures of morphological knowledge, such as production of base words and derived forms. Results revealed both qualitative and quantitative differences between adult struggling readers and the control groups. Specifically, the group with low literacy lacked sophistication in word reading skills, even for basic words. That finding led authors to recommend that “remediation of college students with learning/reading disabilities should likely begin at this basic word level, and instruction aimed at this level should go beyond grapheme-phoneme correspondence to include the morphological structure of words” (Leong, 1999, p. 236). Similarly, Deacon, Parrila and Kirby (2006) asked post-secondary students with and without histories of reading disabilities to complete lexical decision tasks for derived and pseudo-derived words. Typical readers read true derived forms faster than they read pseudo-derived forms; in contrast, adults with histories of reading disabilities showed no such benefit in reading real derived forms over pseudo-derived forms. Thus, the adults with histories of poor reading skills demonstrated a lack of sensitivity to derivational processing.

### Rationale for the current study’s interventions

Half of the participants received novel instruction in semantic mapping with morpho-phonemic analysis (SM-MPA) that implemented five elements of evidence-based practice: (1) using the principles of effective morphological teaching, (2) creating word sums, (3) studying morphological relatives, and (4) teaching flexible syllable segmentation, and 5) assigning primary syllable stress. Using the principles of effective morphological teaching for adolescents included teaching morphology within the context of rich vocabulary instruction, teaching a cognitive strategy such as hypothesizing about unfamiliar word meanings based on known morphemes, teaching morphemes systematically, and teaching words within meaningful contexts (Kieffer & Lesaux, 2010). Creating word sums (e.g., *please* + *ant* + *ly* = *pleasantly*) has resulted in large learning gains through morphemic analysis and synthesis (Bowers & Cooke, 2012; Bowers & Kirby, 2010; Bowers et al., 2010). In prior research, participants have read morphological relatives (e.g., words that share the same base, like *professor*-*profession*) faster than they have read morphologically unrelated words (Nagy, Anderson, Schommer, Scott, & Stallman, 1989). Teaching flexible syllable segmentation to adolescent struggling readers has resulted in greater learning transfer than teaching whole word study (Bhattacharya & Ehri, 2004). Finally, sensitivity to syllable stress has predicted reading achievement in children (Holliman, Wood, & Sheehy, 2010; Jarmulowicz, Taran, & Hay, 2007).

Half of the participants received the more traditional vocabulary instruction, semantic mapping with whole word study (SM-WWS) that included three elements of evidence-based vocabulary instruction: (1) information about definitions and sentence contexts, (2) multiple exposures to vocabulary words, and (3) student engagement in deep processing of words’ meanings (Baumann, Kame’enui, & Ash, 2003; Beck & McKeown, 2004; Blachowicz & Fisher, 2000). Beck and McKeown (2004) used an intervention program called Elements of Reading^®^: Vocabulary (EOR-V) that delivered all three elements of effective vocabulary instruction to K-5 students from low income school districts, resulting in significant language learning gains. In a large randomized control study, teachers implemented the EOR-V program with primary and intermediate students to supplement their regular literacy curriculum. They taught sophisticated vocabulary words that occurred across multiple content area subjects, called Tier 2 vocabulary. Weekly vocabulary units included reading aloud, viewing photos depicting the words, and discussing specific uses of words in sentence contexts. Students who received the EOR-V instruction made significantly greater gains on tests of word knowledge than did those who received only the regular literacy instruction (Apthorp et al., 2011).

Research also demonstrates the value of multi-sensory spelling instruction to support component literacy skills (Cunningham & Stanovich, 1990; Hulme & Bradley, 1984) as spelling requires integration of visual-motor, kinesthetic and linguistic processes (Graham & Weintraub, 1996). With regard to adult struggling readers and the importance of spelling, a recent analysis of the literacy skills of high-risk young adults found that 44% of the variance in their reading performance was explained by one component comprised of spelling, word reading and decoding together (Mellard, Woods & McJunkin, 2017).

### Research questions

The central question was whether semantic mapping with morpho-phonemic analysis would lead to greater gains in component literacy skills (word attack, word recognition, vocabulary, spelling and comprehension) than semantic mapping with whole word study for adult struggling readers. Based on the results of prior literacy studies with older students, Ehri’s (1999, 2005) sight word theory and Perfetti and Hart’s (2001, 2002) Lexical Quality Hypothesis, two predictions were made. First, it was hypothesized that adults taught morpho-phonemic analysis would make greater gains in component literacy gains for the 40 target words than those taught whole word study. Second, it was predicted that participants given morpho-phonemic analysis would also demonstrate greater gains on standardized tests of reading and language, than those taught traditional whole word study.

## Method

### Participants

The present study was carried out with 34 GED students who were minority language learners, including bilingual Spanish speakers who learned English in childhood (see “Bilingual” in Table 1), and native English speakers (see “Monolingual” in Table 1). Participants were recruited from GED classes at an adult learning center in New York City and paid a modest stipend, just above the legal minimum wage, to participate. They met the following criteria for inclusion: (1) enrollment in or recent completion of a GED program, (2) proficiency in English, (3) age of 18–31 years, (4) at least average nonverbal intelligence, and (5) no reported history of cognitive, neurological, hearing or uncorrected vision problems. Table 1 presents descriptive statistics regarding the demographics of the participants, pretest scores, and analyses of variance between the two treatment groups.

**Table 1:** Characteristics of participants and pre-test standard scores (*N*= 34; 17 per group)

	MPA intervention	WWS intervention	*F*(1,33)	*p*
	*M (SD)*	*M (SD)*		
Age (19–31)	24.65 (3.97)	24.53 (4.53)	0.01	.94
Grade completed (8–12)	10.06 (.97)	10.31 (1.14)	0.48	.49
Mono (M); biling. (B)	(M = 7; B = 10)	(M = 8; B = 9)		
Gender (female; male)	(F = 7; M = 10)	(F = 12; M = 5)		
race (Latino, Afr. descent)	(L = 10; AD = 7)	(L = 9; AD = 8)		
Oral Lang. Prof. (WMLS-R)				
WMLS-R standard score (TONI-4)	80.29 (7.50)	75.18 (13.38)	1.89	.18
Index	92.00 (8.89)	92.50 (6.71)	0.03	.86
WJ-III reading composite				
Grade equivalency	6.51 (1.61)	6.39 (2.06)	0.03	.86
WJ-III letter word ID SS	85.18 (6.48)	82.94 (11.00)	0.52	.48
WJ-III spelling SS	90.24 (9.58)	85.17 (13.14)	1.65	.21
WJ-III pass. comp. SS	87.29 (8.15)	85.18 (8.52)	0.55	.46
WJ-III word attack SS	85.06 (7.83)	82.71 (13.03)	0.41	.53
WJ-III reading vocab. SS	82.00 (6.83)	82.47 (8.66)	0.02	.86
WJ-III spell sounds SS	85.88 (10.99)	83.47 (10.39)	0.43	.52
WJ-III picture vocab. SS	78.47 (8.65)	76.35 (9.34)	0.47	.50

From the 46 people who began the study, 34 completed the entire 6-week study, 17 in the experimental group and 17 in the control group. One third (4/12) of those who withdrew from the study did so before they had started the tutoring phase; two were disqualified when they did not demonstrate English proficiency. Participants reported that their reasons for withdrawal from the study included difficulties with childcare coverage, scheduling, health and transportation.

### Study design

This randomized control study included pretest, intervention and post-test phases. Participants completed pretests to: (1) measure nonverbal intelligence, (2) measure English proficiency, (3) calculate Reading Composites for random assignment, and (4) establish baseline skill levels in reading and language. All testing and intervention sessions were administered individually. To ensure roughly equivalent skill levels for each intervention group, participants were ranked according to their Reading Composite levels which were the average grade equivalency scores on the WJ-III Letter Word ID, Reading Vocabulary, and Passage Comprehension. Participants were matched according to their language learning backgrounds (bilingual and monolingual) and Reading Composite levels, then randomly assigned to one of the two interventions. The balance of treatment groups, with regard to language learning backgrounds and reading levels, was maintained throughout the study, even after attrition.

### Assessments

#### Screenings

To screen for nonverbal intelligence, participants followed oral directions requiring them to choose pictures that completed visual patterns on the Test of Nonverbal Intelligence-4 (TONI-4) (Johnsen, Brown, & Sherbenou, 2010). To screen for English proficiency, they completed two subtests (Picture Naming and Verbal Analogies) comprising the oral language cluster of the Woodcock-Munoz Language Survey- R (WMLS-R) (Schrank, Alvarado, Wendling, & Woodcock, 2009). For Picture Naming, participants followed oral directions requiring them to name pictures, and for Verbal Analogies, they followed oral directions to supply missing words in spoken analogies.

#### Pretests and posttests during training sessions

At the beginning and end of each teaching session, participants completed five informal assessments to measure their learning gains for the target words: (1) Read Words: To measure learning gains in word recognition, participants read target words aloud. For example, they read the word *discrimination* before and after reading a passage about how the civil rights movement fought to end racial discrimination. Inter-item correlation coefficient was .88. (2) Extract Base Words: To measure learning gains in word analysis, participants were instructed to “Circle the main part (root or base) of each word”. For example, they circled the base word *equal* in the word *equality*. Inter-item reliability was .83. (3) Spell Words: To measure learning gains in spelling, participants were asked to spell the target words. For example, they spelled the word *constitutionality* before and after reading about the constitutional amendments. Inter-item reliability was .93. (4) Match Vocabulary: To assess learning gains in vocabulary, participants were instructed to “Match vocabulary words to their meanings” before and after each session. For example, the word *destiny* was matched with *purpose or fate*. Inter-item reliability was .70. (5) Complete Sentences: To measure learning gains in reading comprehension, participants were instructed to choose the words from each set of 10 target words that best completed the sentences. For example, to complete the sentence “The government of the United States works best when people have a ________ to community service,” the correct response was *commitment.* Inter-item reliability was .72. All reliabilities were calculated using a parallel form assumption.

#### Pretest and posttest measures

To measure pretest and posttest performance, participants completed language and literacy tests from the Woodcock Johnson Tests of Achievement-III (WJ-III) (Woodcock, McGrew & Mather, 2001, 2007). Examiners administered Form A at pretest and Form B at posttest for seven WJ-III (ACH) subtests, with the following task requirements: (1) Letter-Word Identification: Read aloud printed words, (2) Word Attack: Read aloud pseudowords, (3) Spelling Dictation: Spell words to dictation, (4) Spelling of Sounds: Spell pseudowords to dictation, (5) Reading Vocabulary: Read vocabulary words aloud, produce synonyms or antonyms, and complete verbal analogies, (6) Picture Vocabulary: Name pictures of animals, places and other categories depicted, and (7) Passage Comprehension: Supply missing words to complete sentences and passages after silent reading.

### Interventions

Both interventions taught the same 40 morphologically complex words embedded within civics passages. Each participant had a total of 6 individual sessions totaling about 12 h over 6 weeks, including 2 h of pre-testing (week 1), 8 h of individual tutoring (weeks 2, 3, 4, 5), and 2 h of post-testing (week 6).

The 40 vocabulary words, selected from a high school civics text, were chosen to comply with the characteristics of academic vocabulary, mostly nouns derived from Latin and Greek word origins (Nagy & Townsend, 2012). Low frequency target words were selected so they would be less familiar to participants. Target words were submitted to the English Lexicon Project database (Balota et al., 2007) for an analysis of their attributes. All words were: (1) morphologically complex, with at least one base word and affix; (2) low-frequency words, occurring not more than 25 times per million in a spoken word index, and not more than 30 times per million in a printed words index. Selected words ranged from 2 morphemes (e.g., *citizen* + *ry* = *citizenry*) to 4 morphemes (*abolish* + *ion* + *ist* + *s* = *abolitionists)*. Definitions, synonyms, word origins, and morphological relatives were created using online dictionaries and etymology resources.

Academic vocabulary words were taught over 4 weeks (10 words per week) using semantic maps displaying the same synonyms, definitions, first sentence contexts and civics passage contexts as well as the same number of teaching elements for each treatment group. During each tutoring session, participants completed pretesting, instruction, and post-testing for 10 target words given the following tasks: (1) Read Words, (2) Extract Base Words, (3) Spell Words, (4) Match Definitions, and (5) Complete Sentences. Both groups followed the same instructions to read aloud words, definitions, synonyms, first sentence contexts and civics passages about the rights and responsibilities of American citizens from *We the People: The Citizen & The Constitution*, Level 3 (Center for Civic Education, 2009). For each treatment, the tutor and participant took turns reading scripted questions and responses that were color-coded to indicate tutor and student readings, and presented in a PowerPoint slideshow with 580 slides on a laptop computer. Students were given individual binders containing the target word pretests and posttests, and the semantic map worksheets. Each intervention implemented elements of evidence-based literacy practice, as outlined in the Rationale for the Interventions, and was delivered with a multisensory (visual and auditory) and multi-modality (spoken and written) approach.

**Fig. 1:**
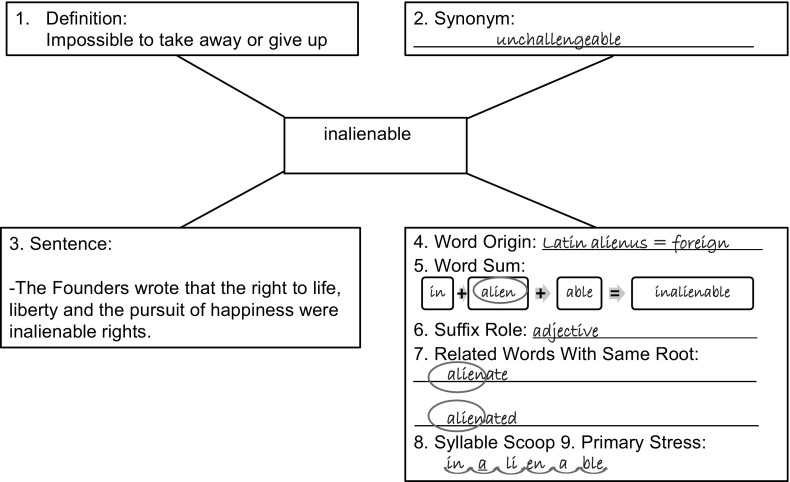
Semantic map with morpho-phonemic analysis (SM-MPA)

Participants assigned to Semantic Mapping with Morpho-Phonemic Analysis (SM-MPA) were taught the 40 academic vocabulary words through sub-lexical (within word) analysis using a semantic map graphic organizer that displayed each of the following teaching elements and participant tasks: (1) Read aloud definition. (2) Read aloud and write the synonym. (3) Read aloud the first sentence context. (4) Read aloud and write the word origin. (5) Read aloud and write the word sum (e.g., virtu/ e + ous = virtuous, with slash preceding the dropped letter). (6) Identify and write the role of the suffix, given a list of parts of speech. (7) Read and write morpohologically related words with the same base word, circling base words, (8) Segment syllables flexibly, drawing scoop lines beneath syllables (Any syllable division with one beat and one vowel sound was acceptable.) and (9) Assign primary stress, underlining the syllable the greatest stress. Corrective feedback was given as necessary (see Fig. 1).

**Fig. 2:**
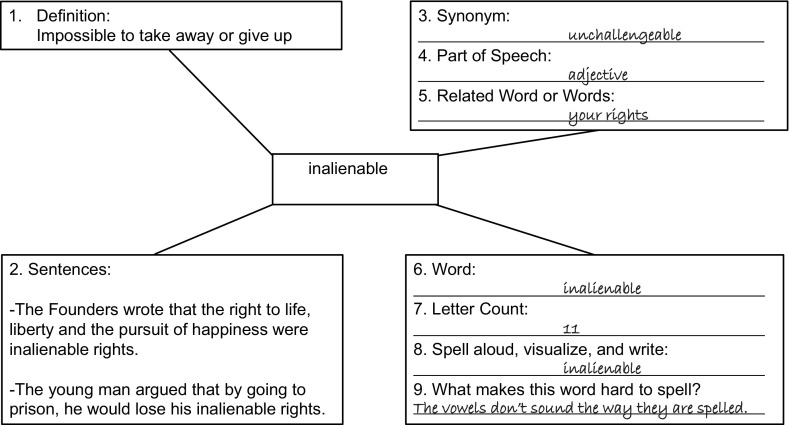
Semantic map with whole word study (SM-WWS)

Students assigned to the control intervention, Semantic Mapping with Whole Word Study (SM-WWS), received traditional evidence-based vocabulary instruction, involving the teaching of whole words without analyzing words’ internal meaning or sound structures. Their semantic maps displayed the following teaching elements and participant tasks: (1) Read aloud definition. (2) Read aloud two sentence contexts, the second of which was about a young adult to increase personal connections for participants. (3) Read aloud and write the synonym. (4) Identify and write the part of speech, given a list of parts of speech. (5) Identify and write a related word, given the question “What does this (target) word make you think of and why?” (6) Read aloud and write the target word. (7) Count and write the number of letters in the word. (8) Spell aloud the letters in the word, visualize the word, then write it again. (9) Perform at metacognitive task given the question “What makes this word hard to spell?” Corrective feedback was given as necessary (see Fig. 2).

### Procedural safeguards and fidelity checks

Four procedural safeguards were implemented to reduce potential teaching and testing bias. First, two tutors, the primary investigator and a research assistant unaware of the research hypothesis, delivered the instruction after randomly assigning matched pairs of participants to treatments. Second, posttests were administered to participants by tutors who had not worked with them; therefore, tutors were blind to the kind of treatment that had been given. Third, tutors followed scripted PowerPoint presentations with controlled parallel elements of instruction. Fourth, scoring of the standardized tests was done using the computer scoring software from the Woodcock-Johnson Tests of Achievement (Woodcock et al., 2001, 2007), then double checked through manual scoring.

To check tutors’ fidelity to scoring procedures, adherence to treatment scripts and protocols, and time spent in treatment, the primary investigator and two research assistants completed checklists to: (1) double score all formal and informal tests; (2) randomly check 25 percent of participant binder content, which included pretests and posttests for the target words and completed semantic maps for each of the 40 target words, and (3) apply ANOVAs for time spent in treatment.

## Results

### Data analyses

Characteristics of participants, Reading Composites in grade equivalencies, and pretest scores on standardized reading and language tests were compared through ANOVAs to determine whether there were group differences prior to treatment. Gain scores were calculated as posttest score minus pretest score. Effect sizes were calculated using Cohen’s *d* (Cohen, 1988) with the following formula: {*d* = posttest *M*-pretest *M*/pretest standard deviation}. Pretest standard deviations were considered more meaningful than pooled standard deviations because they were in the units of the original measurements (Hawell, 2008). ANOVAs were applied to gain scores to compare groups.

Table 1 shows that the two treatment groups did not differ significantly on any demographic or standardized pretest measure. Both groups had reading skills that were estimated to be at the 6th grade equivalency level, as measured by the Reading Composite. Participants’ level of reading competence falls below expectations for GED programs, which cater to students with at least 8th grade level reading skills, although it is comparable to reports of prior studies in which GED students read below the 5th grade level (Perin et al., 2006). In terms of oral language proficiency, both groups had limited to severely limited skills on the WMLS Oral Language Cluster (Picture Vocabulary and Verbal Analogies subtests), consistent with prior studies of severely reduced vocabulary skills in both bilingual and monolingual students from minority groups in low-income urban school settings (Beech & Keys, 1997; Droop & Verhoeven, 2003; Hutchinson et al., 2003; Leseman & de Jong, 1998; Verhoeven, 2000). Both groups had average nonverbal IQ standard scores of 92, indicating average nonverbal intelligence. Participants had a mean age of 24 years, and a mean education level of 10th grade completion. Groups did not differ significantly on languages spoken (monolingual or bilingual) and both groups had slightly more participants of Latina than African American descent.

### Outcome measures for target words

Table 2 shows the gain scores, effect sizes and ANOVAs for the informal assessments of target words. Both groups made large gains on outcome measures of Read Words, Spell Words, Match Definitions, and Complete Sentences, with no significant differences between the groups. However, for the Extract Base Words subtest, gain score differences were highly significant (*p* ≥ .001) with those who received SM-MPA instruction making much larger gains (*d* = 2.67) than those who received SM-WWS (*d* = 0.25).

**Table 2:** Mean pretest, posttest, gain score, effect size, and ANOVA by group for target words

	Pretest	Posttest	Gain	*d*	*F*	*p*
	*M (SD)*	*M (SD)*	*M (SD)*		(1, 32)	
Read words (word recognition) (max = 40)						
SM-WWS	24.00 (10.86)	33.59 (9.04)	9.59 (5.52)	0.88	1.52	.23
SM-MPA	24.94 (8.66)	36.88 (4.31)	11.94 (5.62)	1.38		
Extract base words (word analysis) (max = 40)						
SM-WWS	13.82 (7.62)	15.71 (7.13)	1.89 (4.28)	0.25	35.19	.001***
SM-MPA	20.29 (3.77)	30.35 (3.71)	10.06 (3.73)	2.67		
Spell words (spelling) (max = 40)						
SM-WWS	20.71 (11.95)	29.88 (11.17)	9.17 (5.82)	0.77	1.59	.22
SM-MPA	24.88 (9.47)	32.06 (8.07)	7.18 (3.52)	0.76		
Match definitions (vocabulary) (max = 40)						
SM-WWS	18.59 (7.64)	26.41 (8.24)	7.82 (5.92)	1.02	0.64	.43
SM-MPA	22.31 (6.49)	28.56 (7.80)	6.25 (5.39)	0.96		
Complete sentences (comprehension) (max = 40)						
SM-WWS	19.65 (8.37)	26.53 (9.17)	6.88 (5.63)	0.82	0.00	.98
SM-MPA	22.31 (6.49)	28.56 (7.80)	6.25 (5.39)	0.96		

*Note* *** p < .001

### Outcome measures for standardized reading and language tests

Table 3 shows the gain scores, effect sizes and ANOVA statistics for each group on standardized tests of reading and language. In contrast to the large gains and effect sizes seen for target word assessments, gain scores and effect sizes for the standardized reading and language tests were small or nonexistent. However, ANOVA statistics revealed significant differences on the two standardized subtests that involved complex word reading. Specifically, the experimental group who received morpho-phonemic analysis of target words demonstrated greater gains for both the WJ-III Word Attack (*p* < .04) and Letter Word ID (*p* < .01) than the group who received whole word study, providing evidence of greater learning transfer to untaught words. For all other standardized tests of reading and language, group differences were not significant.

**Table 3:** Mean pretest, posttest, gain score, effect size, and ANOVA by group on standardized reading and language tests (*N* = 34; 17 per group)

	Pretest	Posttest	Gain	*d*	*F*	*p*
	*M (SD)*	*M (SD)*	*M (SD)*		(1, 32)	
WJ-III letter word ID SS						
SM-WWS	82.94 (11.00)	82.12 (10.64)	−.82 (3.75)	−0.07	7.24	.01**
SM-MPA	85.18 (6.48)	86.29 (7.31)	1.12 (2.98)	0.17		
WJ-III spelling SS						
SM-WWS	85.18 (13.14)	85.18 (13.97)	.76 (5.57)	0.00	0.05	.83
SM-MPA	90.24 (9.58)	91.41 (11.16)	1.18 (5.76)	0.12		
WJ-III passage comprehension SS						
SM-WWS	85.18 (8.52)	81.18 (17.78)	−4.0 (3.98)	−0.47	0.77	.39
SM-MPA	87.29 (8.15)	85.82 (8.14)	−1.47 (4.24)	−0.18		
WJ-III word attack SS						
SM-WWS	82.71 (13.03)	81.47 (11.83)	−1.24 (6.90)	−0.10	4.51	.04*
SM-MPA	85.06 (7.83)	88.24 (7.66)	3.18 (5.08)	0.41		
WJ-III reading vocabulary SS						
SM-WWS	82.47 (8.66)	81.18 (8.16)	−1.29 (3.27)	−0.15	1.66	.21
SM-MPA	82.00 (6.83)	82.35 (5.99)	.35 (4.12)	0.05		
WJ-III spell sounds SS						
SM-WWS	83.50 (10.73)	84.75 (8.25)	1.25 (5.00)	0.12	.20	.66
SM-MPA	85.88 (10.99)	86.82 (11.10)	.94 (6.23)	0.09		
WJ-III picture vocabulary SS						
SM-WWS	76.35 (9.34)	74.76 (8.79)	−1.59 (3.45)	−0.17	1.42	.24
SM-MPA	78.47 (8.65)	78.59 (8.87)	.12 (4.78)	0.01		

*Note* ** p < 01; * p < .05

### Fidelity measures

Fidelity checks measuring adherence to scoring procedures and treatment protocols revealed high levels of consistency between tutors for test scoring accuracy and adherence to the treatment protocols, although slightly more time was spent with participants in the SM-WWS condition. All test scores were checked by two independent scorers, with 98% scorer agreement. Due to an irregularity, one participant’s Letter Word ID score was not included in the analysis. Twenty-five percent of the worksheets in participants’ binders were randomly checked by a second person for fidelity to treatment using a rubric of scripted instructional elements. Both groups had a high level of adherence to treatment protocols, with 92% adherence for the SM-WWS group and 98% adherence for the SM-MPA group. Analyses of variance revealed a significant difference in teaching time (*p* < .05) with the SM-MPA group receiving less instruction time on average (*M* = 427 min) than the SM-WWS group (*M* = 454 min).

## Discussion

The first hypothesis, that adult struggling readers who received morpho-phonemic analysis would show significantly greater learning of target words, was supported by only one of the five target word assessment tasks. Unexpectedly, both groups made equally large gains for the Read Words, Spell Words, Match Definitions and Complete Sentences tasks. As expected, the group taught morpho-phonemic analysis made significantly larger gains than the group taught whole word study on the Extract Base Words task. That difference reflected the intervention emphases, as only the SM-MPA group was explicitly taught to circle base words in word sums and morphological relatives.

The second hypothesis, that participants who received morpho-phonemic teaching would demonstrate greater gains on standardized reading and language tests received partial support in the area of word reading. Specifically, participants given morpho-phonemic analysis made significantly greater gains on the WJ-III Letter Word Identification and Word Attack subtests. Letter Word Identification involved reading words, whereas Word Attack involved reading pseudowords. That finding is important because lower level reading skills like word recognition and word attack are among the first linguistic hurdles to clear for successful reading comprehension. If readers are unsuccessful in reading and analyzing words, then they are also likely be unsuccessful in comprehending words and sentences, because higher level skills depend upon accurate, automatic word reading skills (Perfetti, 2007).

Group gains did not differ significantly on standardized measures of vocabulary, spelling, or comprehension, contrary to the second hypothesis. Although the morpho-phonemic group surpassed the whole word group for all standardized measures except the WJ-III Spelling of Sounds subtest, those differences were small and not significant. Group differences must be interpreted with caution given the small number of participants and the small gains in scores. The fact that gains were not greater for vocabulary and comprehension may reflect the short 8 h, 4 week duration of the treatment. These findings may lend support to those of Bowers et al. (2010). Morphological teaching at the sub-lexical layer of word attack, and at the lexical layer of word recognition may tend to produce greater, and perhaps more immediate, gains than morphological teaching at the supra-lexical layer of reading comprehension.

Results of this preliminary study support teaching older readers to use sub-lexical analysis to parse words’ morphemes and syllables, rather than teaching them to study whole words. Participants had greater learning transfer after word analysis. This finding is consistent with Bhattacharya and Ehri’s (2004) study of adolescent struggling readers. They found superior transfer of word learning after instruction in word analysis, compared with a lack of word learning transfer for whole word instruction. Although their study differed from the current study in that it did not include morphological analysis, both studies provided syllable segmentation.

Results of the present study support prior research demonstrating benefits from morpho-phonemic analysis and evidence of morphological compensation for adult readers. In Alamprese et al.’s (2011) study, adults with low to intermediate literacy skills were taught about the morpho-phonemic structure of written English through analysis of phonemes, morphemes and spellings. Those who were taught morpho-phonemic word analysis made greater gains in decoding than those taught the regular adult literacy curriculum. In the current study, adults given structured word analysis showed greater gains not only in decoding, but also in word recognition. With regard to morphological compensation, college students with dyslexia appeared to be using morphological decoding, rather than purely phonological decoding, as an alternative pathway to decoding complex words (Law et al., 2015). Similarly, the participants in the current study may have utilized their newly developed morpho-phonemic decoding strategies to better recognize unfamiliar words.

### Implications for practice

Three reasons why morpho-phonemic instruction may be more effective than whole word instruction are that it is sound from a theoretical perspective, it casts a wider instructional net covering more component literacy skills, and it is a more efficient use of teaching time due to learning transfer.

First, morpho-phonemic literacy instruction is sound from a theoretical perspective with regard to the morpho-phonemic structure of English orthography and theories of word learning and literacy. Ehri’s (1978, 1999, 2005) theory of sight word learning asserts that readers must analyze words' linguistic identities (e.g., meanings, pronunciations and spellings) in order to amalgamate their identities into an orthographic image that is recognized in the lexicon automatically. In the current study, this may have been accomplished as participants learned to analyze constituent morphemes and syllables within words and extract common base words from morphologically related words. Similarly, learning words’ meaning, sound, and spelling connections may have enabled readers to enrich their internal representations of words or lexical representations, thereby increasing fluent word reading skills as theorized in Perfetti and Hart’s (2001, 2002) Lexical Quality Hypothesis. A lengthier morpho-phonemic intervention ought to lead to additional benefits in vocabulary and comprehension, after efficient word identification skills have been achieved (Perfetti, 2007).

Second, teaching poor readers to analyze words’ morpho-phonemic structures casts a wider instructional net to reach the diverse needs of adult struggling readers. Although they may have difficulties with any component of literacy, a large proportion of adults with low literacy skills struggle with decoding and recognizing words, as seen in severe reading disabilities like dyslexia (Greenberg et al., 2011). In addition, a substantial proportion of adults in adult education programs come from minority language backgrounds with vocabulary difficulties (Perin et al., 2006) and adolescent minority language learners have responded well to vocabulary instruction teaching morphemes (Lesaux et al., 2010). In the current study, although both interventions focused on vocabulary, morpho-phonemic analysis concurrently addressed students’ needs in decoding, resulting in gains in both word attack and word recognition.

Finally, teaching morpho-phonemic analysis appears to be a more efficient use of teaching time than whole word study because it leads to learning transfer. In the current study, the morpho-phonemic group actually received significantly less teaching time, yet they succeeded in reading more unfamiliar words after learning to analyze morphemes, the building blocks of all words. In contrast, those provided with whole word instruction learned to read only the words that they were directly taught, necessitating word by word instruction at too slow a pace for adult struggling readers to catch up and attain proficient literacy skills.

### Strengths and limitations

The study was strong in its design to minimize threats to internal validity. Participants completed screenings in nonverbal intelligence and English proficiency. Testers were blind to the treatment when they conducted the standardized posttesting and research assistants were not made aware of the hypotheses being tested. Controlled instruction further reduced threats to validity, with parallel teaching elements and structured semantic maps for each treatment condition. Instructional elements were reportedly clear to both participants and tutors. Differences between treatments were easily distinguishable, both visually and verbally, to enhance teaching clarity. Teaching scripts were presented via PowerPoint presentations, that were color coded to indicate which sections were to be read aloud by the tutors and which were to be read aloud by the participants. Finally, fidelity checks measured high levels of adherence to scoring procedures and treatment protocols. All tests were scored by two independent scorers, with an inter-scorer reliability rate of 98%. Adherence to treatment protocols ranged from 92 to 98%, based on a sampling of 25% of participants’ workbook binders. Only the analysis of teaching time showed significant differences between the groups, favoring the SM-WWS group.

The study was limited by its sampling procedures and design in several ways. First, results may not accurately represent the population of young adults in urban secondary education because participants were volunteers who reported in the debriefing interview that they had participated in order to be paid and to improve their literacy and vocabulary skills. Therefore, they may have represented a lower socioeconomic status group with a greater incidence of reading disabilities than typical GED students. The study was also limited by its sample size, duration and group comparisons. If there had been a larger participant sample who had received the treatment for a longer period than only 8 h, the benefits of instruction may have been more robust for component reading skills. Having a second control group who only received instruction through the GED program would have also been informative. Follow-up studies ought to include a pretest and posttest containing a set of morphologically complex words that were different words from the training words, but similar in terms of their affixes and complexity, in order to isolate the ability to analyze words morphologically, the underlying skill that was being trained.

Another potential criticism of the study is that the SM-MPA group had more practice reading lengthier complex words during the reading of morphological relatives portion of the treatment. However, the SM-WWS group was actually required to read a greater number of morphologically complex words within the second sentence context, although those words were presented within facilitative sentence contexts and were less complex. Specifically, the SM-MPA group was required to read 80 complex words during the morphological relatives portion, with a mean morpheme length of 2.4 morphemes, whereas the SM-WWS group was required to read 201 complex words during the second sentence context portion, with a mean morpheme length of 2.0 morphemes.

### Future directions for morpho-phonemic instruction

Future studies are needed to pinpoint which instructional elements of morphophonemic instruction are most effective for adult struggling readers. Although it appeared that the instruction in word origins, word sums, morphologically related words, flexible syllable segmentation and assignment of primary stress were highly effective, no attempt was made to isolate the separate elements of the teaching to determine which ones were most effective. One of the most effective elements of the treatment may have been the assignment of primary syllable stress, leading to greater accuracy in the pronunciation of complex words (Jarmulowicz, Hay, & Taran, 2008). Further studies are needed to investigate how to teach adult struggling readers to read complex words whose relationships to derived forms are less transparent, such as words whose pronunciations or spellings change from base words to derived forms (e.g., the vowel changes from *aspire* to *aspirations*, or the spelling change from *abolish* to *abolitionists*).

## Conclusions

Few randomized control studies have investigated interventions for adult struggling readers. This experiment compared the impact of morpho-phonemic and whole word vocabulary instruction on component literacy skills of adults with sixth grade level reading skills on average. Greater word reading gains were found after readers were taught to analyze word origins, create word sums with morphemes, extract common base words from morphological relatives, use flexible syllable division and assign primary syllable stress. Results are consistent with theories of word learning and literacy that predict increased reading skills after sub-lexical analysis revealing connections between words’ meaning, sound and spelling structures.

Morpho-phonemic analysis may have been more effective than whole word study in improving word reading skills because it addressed a wider range of component literacy skills. In addition to addressing vocabulary, it concurrently provided instruction in word analysis to address the decoding deficits seen in those with severe reading disabilities like dyslexia. Furthermore, morpho-phonemic analysis appears to have been a more efficient use of teaching time due to greater word learning transfer. Effectiveness and efficiency of instruction are crucial factors when teaching adults who have immense gaps in vocabulary and literacy. They require high impact instruction to become proficient readers, pursue post-secondary education, find meaningful employment, and participate fully in civic life.

## Appendix: Target Academic vocabulary words

**Table:**

Session 1		Session 2		Session 3		Session 4	
(1)	Republicanism	(11)	incriminate	(21)	suffragists	(31)	destiny
(2)	virtuous	(12)	emancipation	(22)	amendment	(32)	disobedience
(3)	philosophers	(13)	proclamation	(23)	adoption	(33)	nonviolent
(4)	inalienable	(14)	ratified	(24)	justices	(34)	segregated
(5)	intolerant	(15)	drafted	(25)	commonwealths	(35)	constitutionality
(6)	restrictions	(16)	abridges	(26)	adapted	(36)	citizenry
(7)	grievances	(17)	immunities	(27)	sovereignty	(37)	sponsoring
(8)	petitioning	(18)	equality	(28)	enlightened	(38)	environmental
(9)	seizures	(19)	servitude	(29)	prosecution	(39)	commitment
(10)	warrants	(20)	abolitionists	(30)	engagement	(40)	aspirations
